# Anemia

**Published:** 1901-11

**Authors:** Wm. Buchanan Conway

**Affiliations:** President Board of Health, Athens, Ga.


### ANEMIA.

By WM. BUCHANAN CONWAY, M. D.,

President Board of Health, Athens, Ga.

Of the essential elements involved in the life of the animal, as well as the vegetable kingdom, none are more easily disturbed by disease than the circulation. Either burdened as it were with the richness of nutrition and proliferating cells, denoting health, or depleted and wasted, until all the vital forces are stagnated, weakness and emaciation takes place and anemia claims its victim.

The warm sunshine that brings seed-time and harvest may crimson the woods and bring forth fairest flowers, but she whose cheeks are ghastly paled by disease, needs something more to paint them with the rosy blush of health. She is unconscious of her approaching danger; life itself, like some passing cloud, is slowly but surely fading away; and when we see her she is worn, wan and wasted. Her cheeks are no longer tinged with nature's blush—but like

> "An opiate vapor, dewy dim,
> Exhaled from out her golden rim,
> And softly fading, drop by drop
> As the mist upon some mountain top."

No class are exempt from this disease; we find it in the palace and hovel, in the workshop and the factory. The rich and the poor share it alike; especially are they predisposed to it, in the rush and excitement of this progressive age.

Over-work, inanition, indiscretions, lack of exercise, nervous emotions, want of fresh air, unhealthy literature, are all important factors in producing this disease; and more especially is the disease found among over-worked girls and boys in large towns, who are confined from day to day in badly ventilated rooms. Nor would I forget to mention a large number of school children throughout our land who are oft-times deprived of fresh air and sunshine.

Menstrual disturbances and uterine abnormalities often arise from these causes, especially about the time of puberty or the menapause; expressions of some pathological change.

We recognize this disease from two standpoints. One in which the blood itself is acted upon by extraneous causes; and the other in which the blood carriers or cells are defective in themselves.

As to the first, any decrease in the volume of blood, whether traumatic or spontaneous, may cause anemia, as in wounds, haemoptysis, epistaxis and menorrhagia.

The total volume of blood being about *one-twelfth* of the weight of the body, the loss of *one-third* of that amount, at any single bleeding, would cause the loss of life. Where the loss of blood is by a slow process, nature often repairs the damage *pari passu.* Again, there are many cases of anemia brought about by a continuous drain upon the system of the albuminous materials of the blood; as is shown in lactation, cancer of the oesophegus or stomach, rapidly growing tumors, Bright's disease and chronic suppurating sores.

In fevers the toxines found in the secretions disturb the functions of blood making. The virus or syphilis and malaria are potent causes of impoverishment of the blood.

We are told that the corpuscles comprise somewhere in the neighborhood of one-half of the weight of the blood; and just here an account of the corpuscles as to their peculiar properties is important, as they form not only a very essential constituent of the circulating fluid, but play a most important part in pathological conditions. By far the most important ingredient of the red corpuscles is the coloring matter, *hemoglobin;* which is remarkable for its avidity to absorb oxygen, as is shown in the venous blood, while being rapidly carried to the lungs; there becoming aeriated and losing its purple color. Hence the physiological functions of the red globules are not only carriers of iron, but carriers and distributors of oxygen throughout the body. The plasma contains about ninety per cent. of water, holding in solution serum, albumen, salts, etc. Two forms of corpuscles are found to exist, the red and white. About 5,000,000 red corpuscles are found to each cubit millimeter of plasma, and the number of white are from 8 to 15,000,000.

The spleen, red marrow, lymphatic glands and liver are special organs of manufacture and development of these cells as well as of their destruction.

The red cells are carriers of iron, and in anemic patients we find a rapid decrease in their number.

The waste and repair constantly in progress throughout the whole body is especially demonstrated in corpuscle life; and hence the rapid depletion on the one hand, or the multiplication of these carriers on the other, and when abnormally out of proportion, *anemia* or *plethera* is the result. If we exclude cases dependent upon parturition or the puerperal state, we will find a large per cent. of cases among the young.

The approach of the disease is generally slow and insidious, the heart is easily made to palpitate, the muscles lose their plumpness, the face is pale and a general feeling of languor pervades the whole system, the eyelids and gums lose their color, hot flashes with dyspnoea, a feeling of faintness or sinking, the appetite fails, a slight oedema is noticed about the feet and ankles, and there is headache and giddiness, also an increasing indisposition to exercise. The urine is pale and of low specific gravity; alas! "the spirit is willing, but the flesh is weak."

This condition of things when found among young girls is generally considered chlorosis.

In a majority of cases the prognosis is favorable, especially where there is an absence of any organic trouble.

As to treatment. Hygienic and dietetic rules should be laid down and strictly adhered to. As long as digestion is good, and no anorexia or vomiting, or any other dyspeptic symptoms develop, the case will not be as troublesome to treat. In cases arising from torpidity of liver with gastro-enteric complications, small doses of some mercurial, with bitter tonics, nux-vomica, hydrochloric acid, and peptonoids are demanded. In cases where there is a deficiency of *red* corpuscles in the blood, iron is regarded by some as a specific. The lactated, tinct. of iron, reduced iron or iron and manganese, all in large doses. Recent demonstrations show that after the administration of inorganic *salts of iron*, that it is especially found in the *liver*. Iron has also been seen in microscopic sections making its way through the intestinal walls, hence proving that it is absorbed by the intestinal tract. Where the stomach is intolerant of iron, some have found good results from the hypodermic injection of a ten per cent. solution of the *citrate* of *iron* and feeding with red bone marrow. However, the treatment is contra-indicated in anemic patients with the following diseases: hepatic cirrhosis, epistaxis and hemorrhoids.

In defective development of young girls, especially those with uterine derangement, the treatment is directed accordingly, as with iron, aloes, phosphorus, arsenic and strychnine.

In hemorrhagic cases the subcutaneous injection of common salt is found most serviceable. And now, in leaving this subject, would that we could earnestly impress upon the minds of the gentler sex, yes! awaken their minds to the importance of good health, especially guarding them against any imprudence, as they are approaching the time of puberty or the climacteric period, guarding every avenue of danger, that their offspring may be healthy, strong and intellectual. For, in the words of Stanley Hall:

"When I see about me in fields of intellectual attainment and culture in the walks of business, and in family life, so many disasters and tragedies long drawn out, of failing health and collapse of nerve, brain and muscle, I feel that health is the only bulwark upon which everything we prize in intellectual culture and religion, can ever be reared. If a nation swings to excess in sin, it must swing back to weakness and decay."

Therefore our women should be careful of their lives. The future success of our great nation depends upon woman's physical development, her culture, her intellect, her progeny. She should not drop below this standard.

Montgomery has well said:

> "Around her childish hearts are twined
> As round some reverend saint enshrined;
> And following hers the childish feet
> Are led to ideals true and sweet."

